# Impaired differentiation of human induced neural stem cells by *TOR1A* overexpression

**DOI:** 10.1007/s11033-020-05390-x

**Published:** 2020-04-01

**Authors:** Felix Stengel, Franca Vulinovic, Britta Meier, Karen Grütz, Christine Klein, Philipp Capetian

**Affiliations:** 1 Institute of Neurogenetics, University of Lübeck, Lübeck, Germany; 2 Department of Neurology, University Hospital Würzburg, Würzburg, Germany

**Keywords:** Dystonia, DYT1, torsinA, *TOR1A*, Neuronal stem cells, Neuronal differentiation, Inducible expression

## Abstract

DYT-TOR1A is the most common inherited dystonia caused by a three nucleotide (GAG) deletion (dE) in the *TOR1A* gene. Death early after birth and cortical anomalies of the full knockout in rodents underscore its developmental importance. We therefore explored the timed effects of TOR1A-wt and TOR1A-dE during differentiation in a human neural in vitro model. We used lentiviral tet-ON expression of TOR1A-wt and -dE in induced neural stem cells derived from healthy donors. Overexpression was induced during proliferation of neural precursors, during differentiation and after differentiation into mature neurons. Overexpression of both wildtype and mutated protein had no effect on the viability and cell number of neural precursors as well as mature neurons when initiated before or after differentiation. However, if induced during differentiation, overexpression of TOR1A-wt and -dE led to a pronounced reduction of mature neurons in a dose dependent manner. Our data underscores the importance of physiological expression levels of TOR1A as crucial for proper neuronal differentiation. We did not find evidence for a specific impact of the mutated TOR1A on neuronal maturation.

**Electronic supplementary material:**

The online version of this article (10.1007/s11033-020-05390-x) contains supplementary material, which is available to authorized users.

## Introduction

**Fig. 1:**
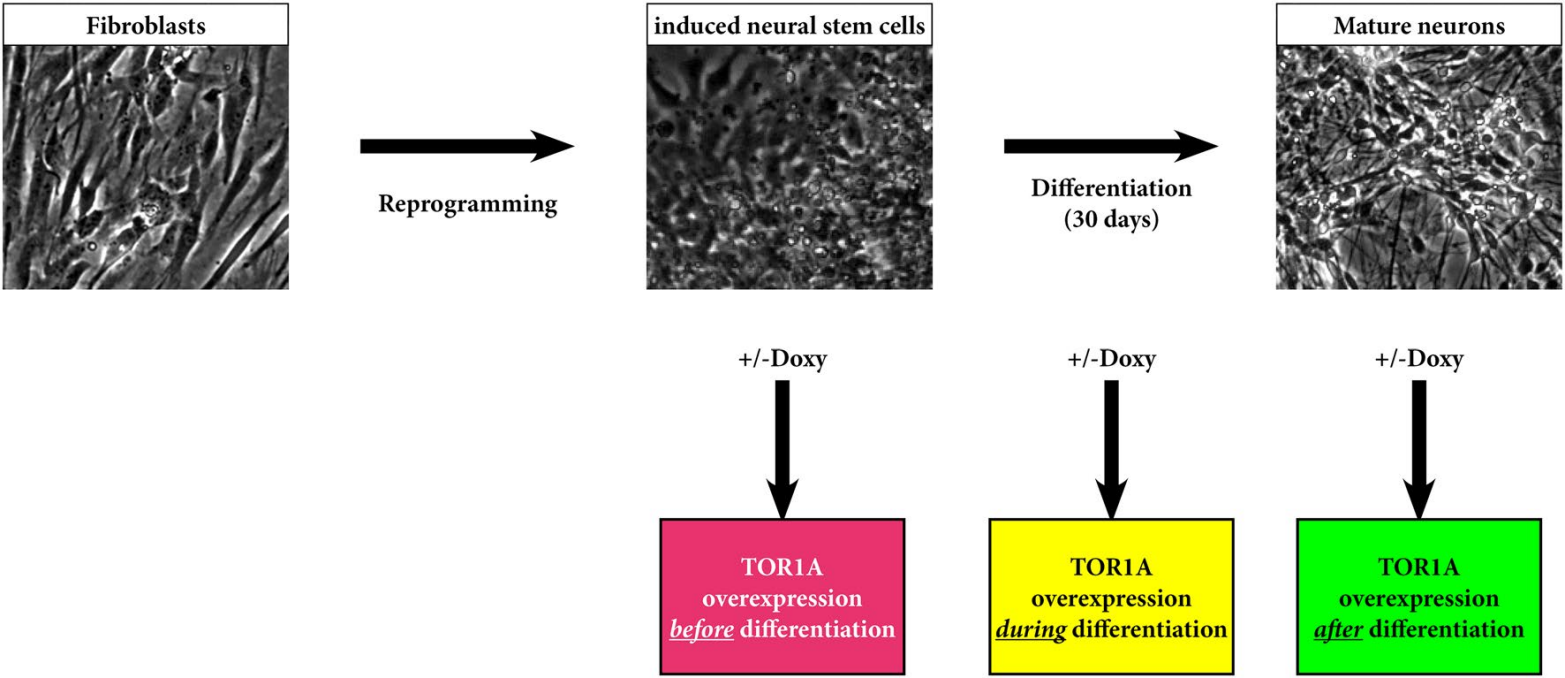
Overview of the performed experiments: After reprogramming and virus transfection of induced neural stem cells (iNSC), overexpression was initiated before, during and after differentiation to mature neurons

DYT-TOR1A is a generalized early onset dystonia with autosomal dominant inheritance caused by mutations in the *TOR1A* gene encoding torsinA (TOR1A). The most common mutation is a three nucleotide in-frame deletion, c.907_909delGAG (dE), leading to a loss of a glutamic acid near the C-terminus [1]. TOR1A functions as an AAA+ chaperone-like protein and resides at the endoplasmatic reticulum and inner nuclear membrane [2]. In several overexpression models the dE mutant torsinA shows perinuclear accumulation and membrane inclusions as opposed to the wildtype protein which is evenly distributed throughout the endoplasmatic reticulum [3, 4]. The role of TOR1A for proper central nervous system (CNS) development (which is undoubtedly only one of many) is underscored by the fact that mice without a wild type allele (−/−, dE/dE or −/dE) die shortly after birth. Thirty percent of full knockout animals (−/−) show anomalies of neural tube closure (although also animals without gross anomalies die shortly after birth). A breakdown of proliferative zones in the ventricle wall due to defects of radial glia polarity leads to excess production of neural material [5]. However, neither the common heterozygous dE mutation [1], nor the much rarer and more severe recessive mutations of *TOR1A* [6] are associated with brain malformations. The goal of our study was to study the effects of overexpressed wildtype and mutant (dE) TOR1A on proliferating human neural stem cells and their differentiation towards mature neurons in vitro. Induced neural stem cells (iNSC) reprogrammed from fibroblast cultures of healthy donors [7] were transduced by a lentiviral inducible tetON system [4]. This allowed us studying the effects of overexpressed protein in a timed manner before, during and after differentiation (Fig. 1).

## Results and discussion

### Robust and inducible overexpression of MYC-tagged torsinA

**Fig. 2:**
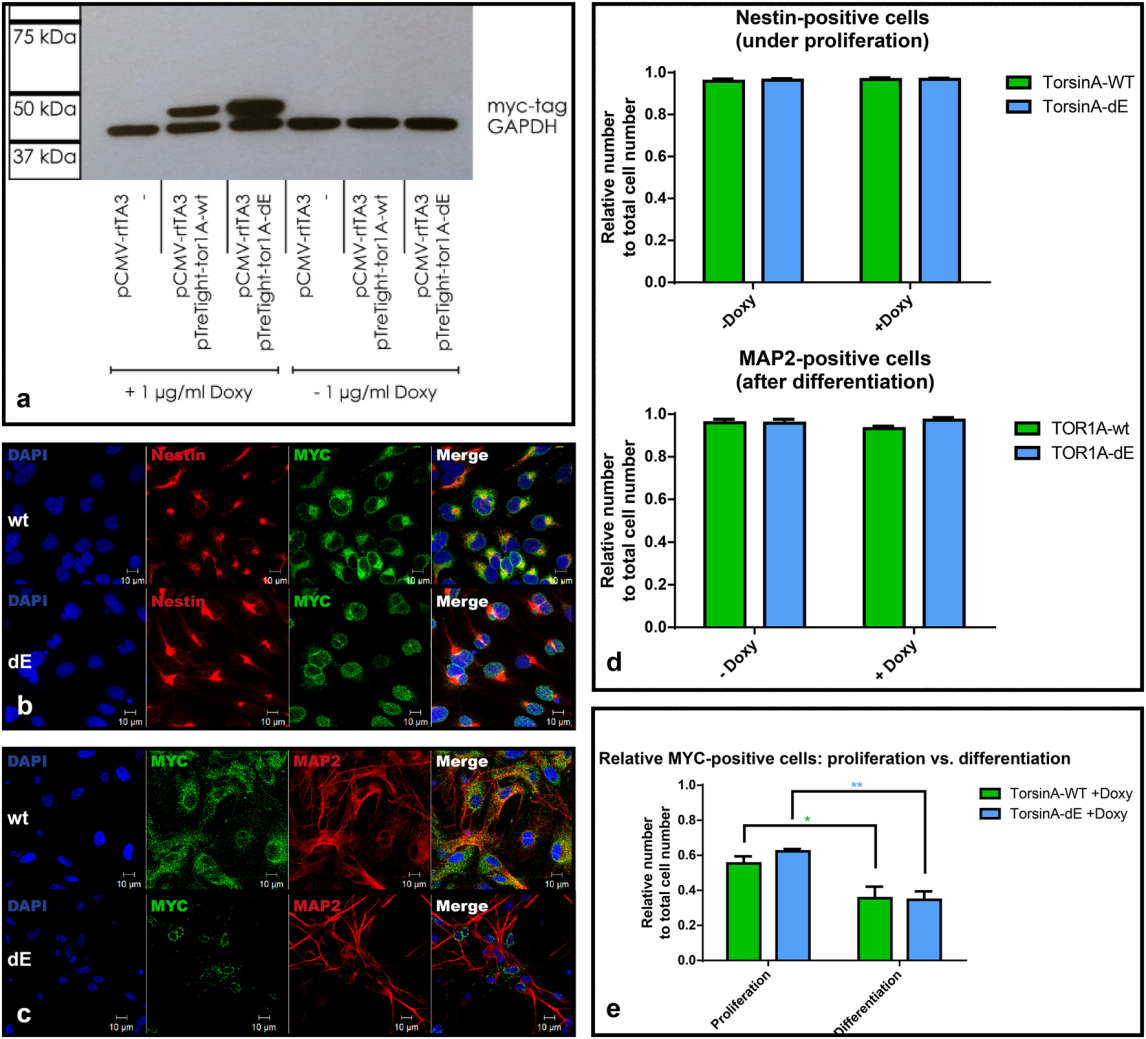
Inducible expression of the transgenic constructs before and after differentiation: Tightly controlled inducible expression of TOR1A-wt and dE in NSCs verified by positive MYC-tag in western blot at 40 kDa. GAPDH was used for loading control and shows a signal at 37 kDa (**a**). Immunofluorescence of iNSC positive for Nestin (red, **b**) and mature neurons positive for MAP2 (red, **c**) after Doxy-induction for 72 h. Localization of the expressed construct is demonstrated by the MYC-tag (green). TOR1A-wt is distributed throughout the entire cell body (**b**, **c** upper row) while TOR1A -dE shows perinuclear aggregation (**b**, **c** lower row). The relative amount of Nestin- and MAP2-positive cells remained unchanged after expression of both TOR1A variants (**d**, n = 3). The relative amount of MYC-positive cells showed a highly significant reduction after differentiation for both TOR1A variants (**e**, two-way ANOVA test, for culturing condition: p-value < 0.0001, error bars represent SEM, n = 3). (Color figure online)

Expression of the tagged wildtype and mutant TOR1A was tightly regulated by the presence of Doxycycline (Doxy) (Fig. 2a). Overexpressed protein showed a characteristic subcellular distribution after 72 h of induced expression both in undifferentiated Nestin-positive neural stem cells as well as mature microtubule associated protein 2 (MAP2)-positive neurons: TOR1A-wt with an even distribution throughout the cell body, TOR1A-dE forming clusters in the perinuclear space (Fig. 2b, c) [3, 8].

Under antibiotic selection for the respective constructs, 55% of all cells expressed TOR1A-wt and 62% TOR1A-dE under proliferative conditions. This amount was significantly reduced after differentiation to 36% for TOR1A-wt and 35% for TOR1A-dE (Fig. 2e). A possible explanation could be the different activity of the CMV promoter [parts of which are a component of the tet responsive element (TRE)] during different stages of neural differentiation [10] or selective silencing of the TRE promoter with a still active PGK-promoter of the antibiotic resistance.

The relative amount of neural stem cells and neurons showed no significant difference between lines expressing the wt or mutated construct (n = 3, two-way ANOVA, p = 0.74 for Nestin and p = 0.27 for MAP2, Fig. 2d).

### TOR1A-overexpression during neuronal maturation

**Fig. 3:**
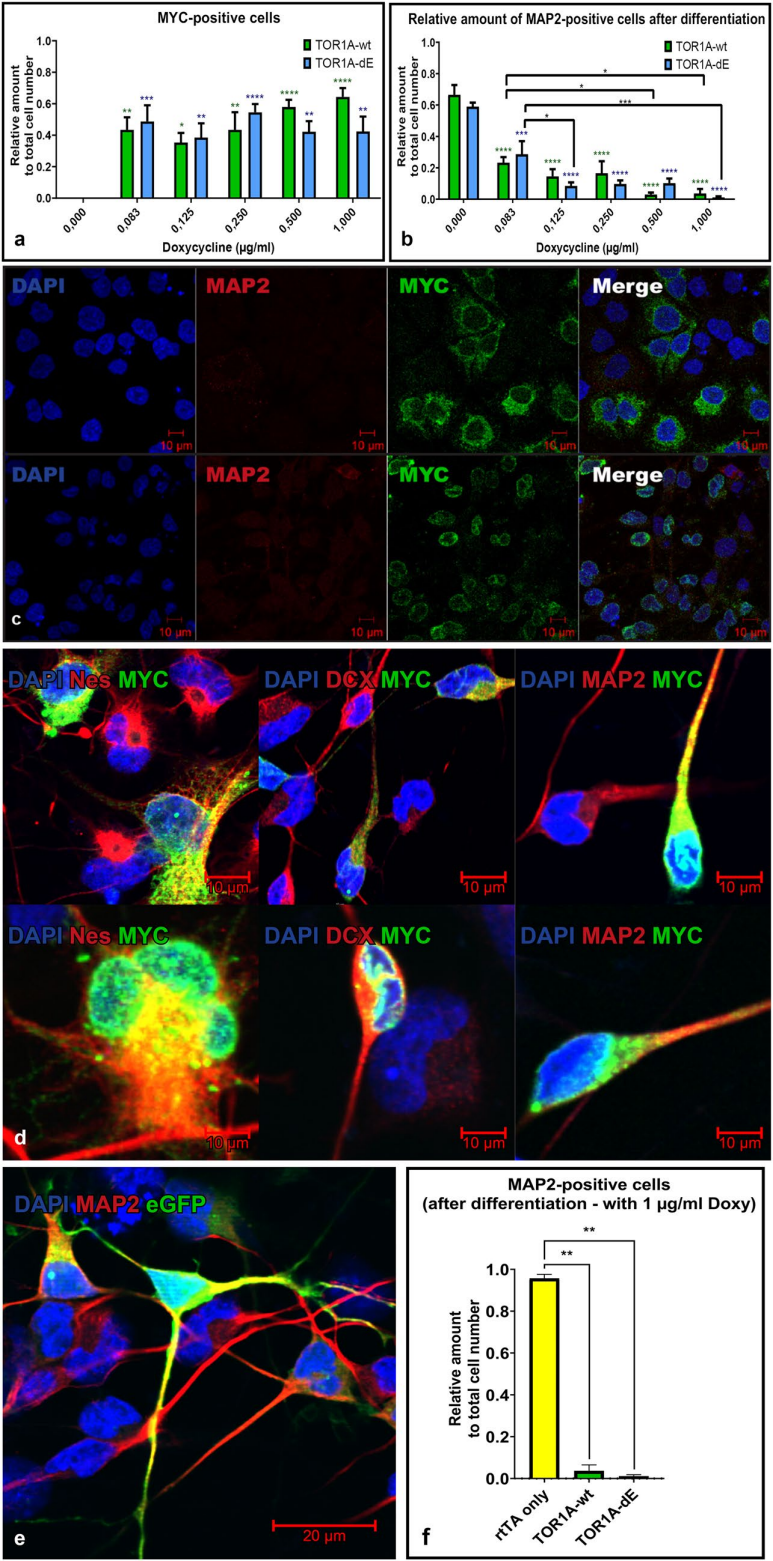
Impact of construct overexpression during neural differentiation: All Doxy-doses demonstrated a significant increase of the relative amount of MYC-tag positive cells but no difference between individual doses (**a**, asterisks referring to control line without Doxy). The relative amount of MAP2-positive cells were reduced in a dose dependent manner after addition of Doxy. All doses showed a significant reduction in comparison to baseline (asterisks over bars). Higher and lower Doxy doses showed a significantly reduced amount of MAP2-positive cells in some cases (b, asterisks over lines,) (**a**, **b**, n = 3). Representative immunofluorescence of cells differentiated in the presence of 1 μg/ml Doxy for 30 days showed the characteristic localization of TOR1A-wt (upper row) and -dE (lower row) by visualization of the MYC-tag (green). No MAP2-positive cells (red) were present (**c**). Cultures fixed at the time of the most pronounced cell death (approximately 1 week after Doxy addition): Overexpressed wildtype (upper panel) and mutated (lower panel) MYC-tagged constructs (green) are present in Nestin (Nes) positive neural stem cells, doublecortin (DCX) positive neuronal progenitors and MAP2 positive mature neurons (all in red) (**d**). Overexpression of eGFP (green) during differentiation for 30 days had no impact on neuronal differentiation to MAP2 positive (red) neurons (**e**). Exposure of Doxy alone had no impact of cells overexpressing only rtTA (Kruskal–Wallis test for multiple comparisons, p = 0.0063 for rtTA vs. TOR1A-wt, p = 0.003 for rtTA vs. TOR1A-dE, n = 3) (**f**). Error bars represent SEM. (Color figure online)

We explored the impact of increasing TOR1A-overexpression on neuronal differentiation by performing a Doxy dilution series with different concentrations. Adding increasing amounts of Doxy to the cell culture medium during differentiation led to a significant increase of the relative number of cells expressing the MYC-tagged construct compared to baseline (n = 3, Tukey’s multiple comparisons test, p < 0.0001) (Fig. 3a). There was no statistical difference between individual concentrations of Doxy (0.083, 0.125, 0.250, 0.500, 1.000 [μg/dl]) and the overexpressed construct (WT and dE). The addition of Doxy is known to increase expression in a dose dependent manner [11] but, understandably, does not change the number of cells expressing the construct per se.

Doxy-induction during differentiation led to a highly significant reduction of the relative amount of MAP2-positive neurons after 30 days in a dose-dependent manner (n = 3, Tukey's multiple comparisons test, p < 0.0001). We observed no differences between the wildtype and mutated TOR1A variant (n = 3, Tukey's multiple comparisons test, p = 0.51) (Fig. 3b).

The immunostaining after 30 days of differentiation (Fig. 3c) demonstrated a nearly complete absence of MAP2-positive neurons at a dosage of 1 μg/mg Doxy. The remaining cells showed the presence of the MYC-tagged construct at the typical intracellular localizations depending on the mutation status.

To determine at what stage of neuronal maturation development arrested and cells died, we tracked cultures during differentiation. At the time point when the majority of cells disappeared from the coverslips (after 5–7 days), coverslips were fixed and stained for the MYC-tagged constructs as well as markers of neural stem cells (Nestin [12]), immature neuroblasts (doublecortin = DCX [13]) and postmitotic neurons (microtubule associated protein 2 = MAP2 [14]). Cells at all stages of development expressed TOR1A-wt as well as –dE constructs (Fig. 3d). However, MAP2 positive neurons overexpressing the TOR1A constructs displayed rather immature features (uni- or bipolar neurites, no ramifications). Thus, cell death must have occurred after differentiation but during maturation of neurons.

The overexpression of the enhanced green fluorescent protein (eGFP) in differentiating iNSC had no impact on cell survival. After 30 days of differentiation, numerous mature neurons co-expressing MAP2 and eGFP were present in culture (Fig. 3e). Thus, we ruled out detrimental effects of protein overexpression as such.

Addition of Doxy to cells overexpressing only rtTA cells in comparison to cells overexpressing rtTA and TOR1A-wt or –dE had no impact on neuronal differentiation (Kruskal–Wallis test for multiple comparisons, p = 0.0063 for rtTA vs. TOR1A-wt, p = 0.003 for rtTA vs. TOR1A-dE, n = 3) (Fig. 3f). We excluded therefore a deleterious impact of Doxy as such.

### BrdU incorporation assay of proliferative iNSC

**Fig. 4:**
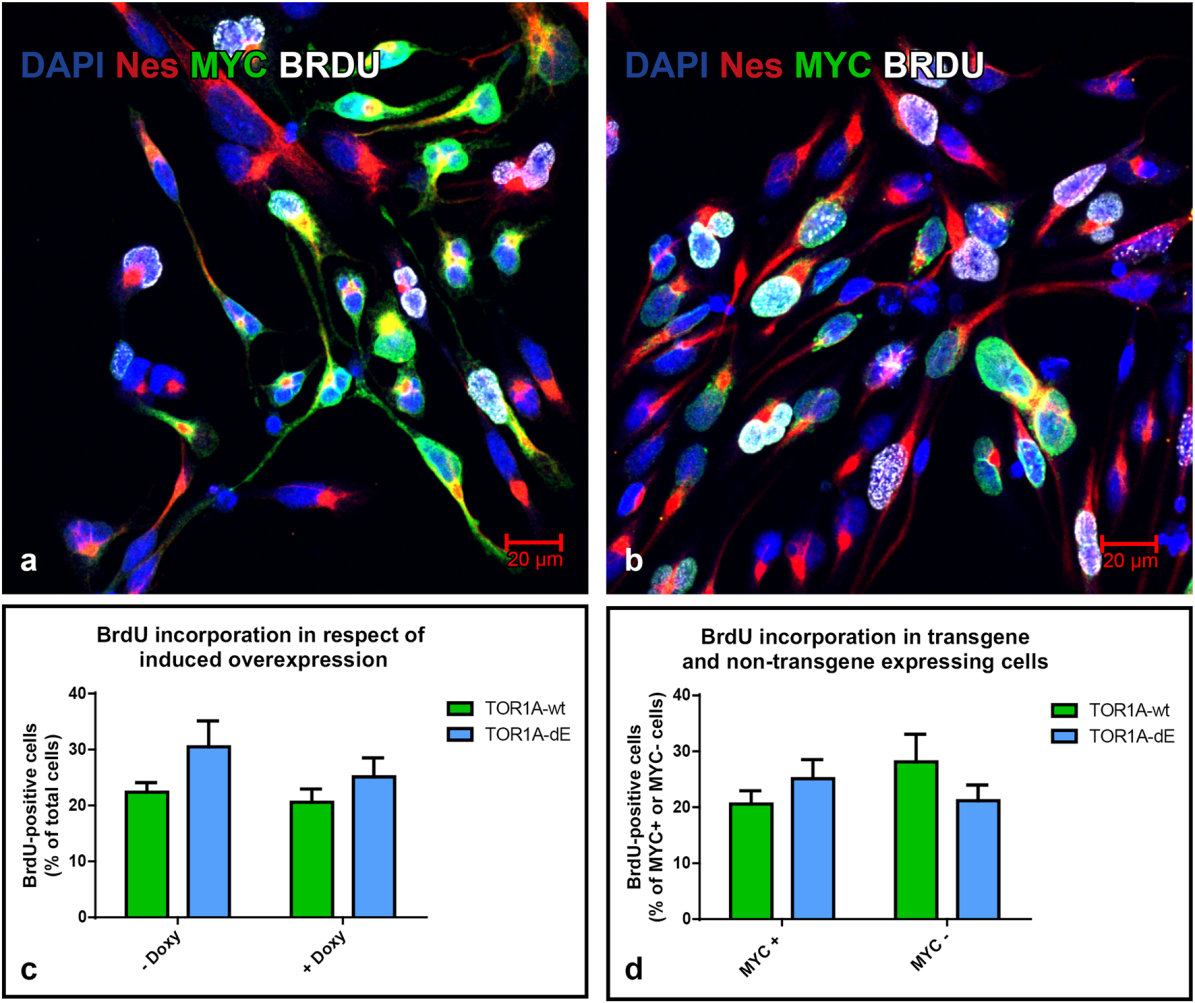
BrdU-incorporation in iNSC under proliferative conditions in respect of transgene expression: Nestin-positive (red) iNSC expressing the MYC-tagged (green) constructs [TOR1A-wt (**a**) and -dE (**b**)] incorporated BrdU (white) in proliferating cells. Induction of the TOR1A-wt as well as -dE construct does not change the percentage of BrdU-incorporating cells in respect to total cell number (**c**). Conversely, after induction, the percentage of BrdU-incorporating cells actually expressing the MYC-tagged construct does not differ to cells not overexpressing the construct (**d**). n = 3, error bars represent SEM. (Color figure online)

It has been shown that the full knockout of TOR1A leads to an excessive proliferation and mislocalization of radial glial cells in a mouse model [5]. We therefore speculated if the overexpression of TOR1A could lead to an increased proliferation of iNSC. This would not become apparent under repetitive passaging of immature cells but lead to overconfluence and detachment during differentiation. Irrespective from overexpression of TOR1A constructs, the amount of Nestin-positive cells under proliferative conditions was considerably higher than 90% (Figs. 2e, 4a and b). We therefore simplified our analysis and determined the relative number of BrdU-positive cells to the total cell number as well as to MYC-tagged cells. Neither the overexpression of TOR1A-wt nor TOR1A-dE led to an increased BrdU incorporation relative to the total cell number or to MYC-positive cells (Fig. 4c and d). Thus, the overexpression of TOR1A does not change the proliferative rate in iNSC and is therefore unlikely to mediate its deleterious effects on neuronal maturation through simple overgrowth in culture.

Overexpression of TOR1A variants during differentiation seems to disrupt neuronal maturation in human induced neural stem cells. Overexpression of both variants disturbs the neuronal differentiation and maturation in a dose dependent manner with higher expression levels of transgene leading to a higher toxicity. The reduction of mature neurons unveils a particular vulnerability of the developing human central nervous system to expressional imbalance of TOR1A. Induced overexpression in proliferating neural precursors and mature neurons have no observable effect. These findings are surprising: Transgenic rodent models demonstrated distinct abnormalities for overexpression of both TOR1A-wt and -dE: increased fiber anisotropy in distinct regions of the brain, subtle behavioral abnormalities and changes in amino acid neurotransmitter levels [15]. However, the cerebral microarchitecture remained unchanged. Furthermore, overexpression by lentiviral infection of primary murine cortical neurons has not been reported to impair differentiation in vitro [16, 17]. Therefore, we think that our findings might represent a specific vulnerability of the maturation of human neural progenitors. Obviously, the negative effect on maturation is not a result of the dE-mutation, but of the increased levels of TOR1A per se. Since the dE-mutation of TOR1A has been shown to exert its detrimental effects through a *loss-of-function* effect [18], animal models carrying a full knock-out of TOR1A have been analyzed and led to further insights through their more severe phenotypes. Similarly as homozygous dE-mutation-carrier animals, these animals also die shortly after birth but show in 30% of all cases a grossly abnormal brain structure with surplus neural material [5]. This could be linked to disorganized proliferative zones with increased numbers of Pax6+ neural precursors due to impaired nuclear migration through an excess of linker of nucleoskeleton and cytoskeleton (LINC) complexes. However, if the effects we observed for overexpression of TOR1A in human neural precursors can be attributed to a similar mechanism seems uncertain: The full knockout lead to an excessive production of neural tissue. A similar occurrence in cell culture would eventually lead to overgrowth, detachment and loss of cells. However, since we did not observe any changes in proliferation of the neural precursors and not only an absolute reduction of cell number but also of the relative amount of MAP2 + neurons, we would rather assume the occurrence of selective cell death during neuronal maturation. The presence of overexpressed and tagged TOR1A in cells of all stages of neuronal differentiation also argues against this. We would therefore rather conclude a particular susceptibility of the maturation of young human neurons to TOR1A expression imbalance. The relevance of these findings for the pathophysiology of DYT-TOR1A remains unclear since no specific effect of the mutation was observed and TOR1A plays several important intracellular roles besides the neurodevelopmental one. Indeed, post-mortem brains from DYT-TOR1A patients exhibited grossly normal cellular architecture of the CNS [19, 20]. This obviously excludes widespread neurodevelopmental abnormalities as a relevant contribution to the pathophysiology of DYT-TOR1A, although subtle changes during neuronal differentiation cannot be studied on post-mortem material. Our study demonstrates that human induced neural stem cells represent a platform for studying neurodevelopmental processes in vitro and could therefore be useful for unrevealing more subtle abnormalities during neuronal differentiation in DYT-TOR1A patient derived cells.

## Material and methods

### Cultivation of iNSC

For our experiments, we used three iNSC lines from healthy volunteers (two clones from one individual, one from another). An overview of the performed experiments is illustrated in Fig. 1. All experiments have been approved by the ethics board of the University of Lübeck. The reprogramming and characterization of the lines has been described before in detail [7]. In short: Fibroblast lines from healthy volunteers were transfected by electroporation with three reprogramming plasmids, expressing five transcription factors (Oct3/4, Sox2, Klf4, Lin28 and L-myc) and a short hairpin RNA directed against p53 [21]. Stepwise addition of neural induction medium (Stemcell Technologies, Vancouver, Canada) lead to the emergence of neural colonies, which were picked and plated on BD Matrigel coated cell culture wells (Corning, USA). Established lines were kept under proliferative conditions with Neural Progenitor Medium (Stemcell Technologies, Vancouver, Canada) and passaged regularly with Accutase (Gibco® by Life Technologies, Carlsbad, USA).

Cells for immunostaining were plated on poly-d-lysine (Sigma-Aldrich, St. Louis, USA) and laminin (Roche, Basel, Switzerland) coated glass coverslips (Karl Hecht, Sondheim, Germany). All cells were maintained in an incubator at 37 °C and 5% CO_2_ (Heracell 150i, Thermo Fisher Scientific, Waltham, USA).

### Lentiviral production

Generation of constructs for induced overexpression by the tet-on system were described before in detail [4]. A reverse tetracycline-controlled transactivator (rtTA) (pLenti CMV rtTA3 Hygro (w785-1), a gift from Eric Campeau, Addgene plasmid #26,730; https://n2t.net/addgene:26730; RRID:Addgene_26730) was employed for inducible expression. This plasmid contained a hygromycin resistance gene under the control of the constitutively active phosphoglycerate kinase (PGK) promoter Two other lentiviral vectors carried the sequence of TOR1A (wildtype and the dE mutation (c.907_909delGAG)) under the control of a pTreTight promoter (Clontech, Takara Bio USA, Inc., USA) and a myc-tag at the 3′ end. These plasmids carried a puromycin resistance gene under the control of a PGK promoter.

HEK293T cells (Life Technologies) cultivated in Dulbecco’s modified Eagle’s medium (PAA Laboratories, Little Chalfont, UK) supplemented with 10% fetal bovine serum (PAA Laboratories) and 1% penicillin and streptomycin (PAA Laboratories), were transfected with the lentiviral plasmids together with the packaging plasmid (pCMV delta R8.2, a gift from Didier Trono, Addgene plasmid #12,263; https://n2t.net/addgene:12263; RRID:Addgene_12263) and the envelope plasmid (pMD2.G, a gift from Didier Trono, Addgene plasmid #12,259; https://n2t.net/addgene:12259; RRID:Addgene_12259) by lipofection (FuGENE HD, Fugent, Columbus, USA). Supernatant containing the lentiviral particles was collected and sedimented by ultracentrifugation at 20 000 rounds per minute (rpm) for two hours in an Optima L-80 xP Ultracentrifuge (Beckman Coulter, Brea, USA). The lentiviral particles were resuspended in OptiMEM (Gibco by Life Technologies, Carlsbad, USA) and stored at − 80 °C. For titering, HEK293T cells were transduced, DNA collected (DNeasy, QIAGEN, Hilden, Germany) and a qPCR with specific primer pairs for a part of the lentiviral sequence as well as the genomic sequence of GAPDH performed (Santa Cruz Biotechnology, Dallas, USA). The multiplicity of infection (MOI) was calculated by the formula 1.

1 $$\mathrm{M}\mathrm{O}\mathrm{I}= \frac{{\mathrm{V}\mathrm{o}\mathrm{l}\mathrm{u}\mathrm{m}\mathrm{e}}_{\mathrm{V}\mathrm{i}\mathrm{r}\mathrm{u}\mathrm{s}}\times{ \mathrm{C}\mathrm{o}\mathrm{n}\mathrm{c}\mathrm{e}\mathrm{n}\mathrm{t}\mathrm{r}\mathrm{a}\mathrm{t}\mathrm{i}\mathrm{o}\mathrm{n}}_{\mathrm{V}\mathrm{i}\mathrm{r}\mathrm{u}\mathrm{s}}}{{\mathrm{V}\mathrm{o}\mathrm{l}\mathrm{u}\mathrm{m}\mathrm{e}}_{\mathrm{C}\mathrm{e}\mathrm{l}\mathrm{l}\mathrm{s} }\times{\mathrm{C}\mathrm{o}\mathrm{n}\mathrm{c}\mathrm{e}\mathrm{n}\mathrm{t}\mathrm{r}\mathrm{a}\mathrm{t}\mathrm{i}\mathrm{o}\mathrm{n}}_{\mathrm{C}\mathrm{e}\mathrm{l}\mathrm{l}\mathrm{s}}}$$

### Lentiviral transduction of iNSC and generation of cell lines

Appropriate concentrations of selection antibiotics were determined beforehand by a kill curve on non-transduced cells (500 μg/ml for hygromycin and 500 ng/ml for puromycin). INSC lines were transduced by lentiviral constructs of pCMV-rtTA3 with a MOI of 0.1. After 48 h, cells were passaged on a new plate and hygromycin for selection was applied to the culture. The emerging cell lines were passaged and transduced a second time with the pTreTight-TOR1A-wt and -dE constructs with a MOI of 0.1. The emerging lines were cultivated further under the continued addition of hygromycin and puromycin.

### Transfection of eGFP constructs in differentiating cell lines

500 thousand iNSC per line were transfected with 2 µg of eGFP control plasmid by electroporation with a *Nucleofector* device [program A-033, Neural Stem Cell Kit (mouse), Lonza, Basel Switzerland] and immediately plated on Matrigel in a six well dish. After 2 days, cells were detached and plated for differentiation as described before.

### Neuronal differentiation and induction of TOR1A expression

For neuronal differentiation of iNSCs, we changed the Neural Progenitor Medium to N2B27 medium (DMEM/F12:Neurobasal 1:1, 1% N2, 2% B27, Gibco by Life Technologies, Vancouver, Canada) and added 20 ng/ml brain-derived neurotrophic factor (BDNF), 10 ng/ml glial cell line-derived neurotrophic factor (GDNF), 10 ng/ml insulin-like growth factor 1 (IGF-1) (all from PeproTech, NY, USA), 0,5 mM dibutyril cyclic adenosine-monophosphate (dbcAMP, EnzoLife Sciences, Farmingdale, USA) and 10 μM of the Notch-pathway inhibitor DAPT (Tocris, Ellisville, USA) for 30 days. TOR1A overexpression was induced by 1 µg/ml Doxy (Sigma-Aldrich, Saint Louis, USA). For Doxy dilution series, Doxy was added to the medium in five different concentrations (0.083 μg/ml, 0,125 μg/ml, 0.25 μg/ml, 0.5 μg/ml, 1 μg/ml) during the neuronal differentiation.

### Immunofluorescence and confocal Imaging

Cells for immunostaining were fixed by 4% paraformaldehyde (PFA) (Merck, Darmstadt, Germany) for 30 min at room temperature (RT). Unspecific epitopes were blocked by incubation in 5% donkey or goat serum in blocking buffer (see Supplemental Table 1 for the composition of the staining buffers) for 45 min at RT. Afterwards, cells were incubated directly with the first antibody in incubation buffer over night at 4 °C (see Supplemental Table 2 for the employed antibodies). Cells were washed three times with washing buffer before incubating the secondary antibodies in dark for 120 min at RT. After three more rounds of washing, auto fluorescence was quenched by incubating with sudan black 0.1% in 70% ethanol for 5 min, followed by two rounds of washing buffer and one in PBS. Coverslips were mounted on glass slides (Menzel Gläser, Braunschweig, Germany) with Vectashield containing DAPI (4′,6-Diamidin-2-phenylindol) (Thermo Fisher Scientific, Waltham, USA). Immunostainings were imaged with a LSM 710 confocal laser scanning microscope (Zeiss, Jena, Germany) and ZEN black software (Zeiss, Jena, Germany). Eight representative visual fields (134.8 μm × 134.8 μm) were taken of every staining for quantitative analysis. Cells were counted manually by a blinded observer by the ImageJ software (NIH, Bethesda, USA).

### BrdU incorporation assay

INSC were plated on coated coverslips (25.000 cells/coverslip) as described before. This cell number was determined beforehand and ascertained that cells did not reach full confluency until the end of the experiment. After 48 h, induction of transgene expression was induced by the addition of 1 µg/ml Doxy. After another 72 h, BrdU (Sigma-Aldrich, St. Louis, USA) was added to final concentration of 10 µM to the cells and incubated for 3 h at 37 °C. Cells were fixed as described before. DNA hydrolysis was performed by addition of 2 N HCl (Carl Roth, Karlsruhe, Germany) for 30 min at 37 °C. After washing the cells with PBS, immunofluorescent staining against BrdU in combination with Nestin and Myc was performed as described before.

### Protein assays

Protein expression was determined by Western Blot. For protein extraction, cells were lysed with RIPA buffer including protease inhibitor and phosphatase inhibitor detergent (see Supplemental Table 1 for the buffer composition) at 4 °C for 35 min followed by centrifugation at 13,000 rpm for 20 min. By bicinchoninic acid (BCA) protein assay, concentration of proteins was determined. We balanced protein concentration to 10 μg per sample. Adding loading buffer (see Supplemental Table 1 for buffer composition) as well as reduction agent Dithiothreitol (DTT) (Sigma-Aldrich, St. Louis, USA), proteins were denaturized at 95 °C for 4 min. Sodium dodecyl sulfate polyacrylamide gel electrophoresis (SDS-PAGE) was started with 100 V (V) for ten minutes. Once the samples entered the separation gel, voltage was increased to 150 V for about 60 min. The transfer on polyvinylidene fluoride (PVDF) membranes (Merck, Darmstadt, Germany) was carried out at 32 V for one hour. We used 5% skim milk in Tris-buffered saline with Tween20 (TBST) for blocking unspecific epitopes for one hour. Primary antibodies were incubated at 4 °C for 12 h. By washing the membrane three times for 15 min with TBST unattached antibodies were removed. Secondary antibodies in 5% skim milk in TBST were incubated for two hours at RT and subsequently membranes were again washed by TBST. Membranes were incubated with enhanced chemiluminescence (ECL) mix for two minutes before exposing a regular photosensitive film (GE Healthcare Limited, UK). The density of the bands was quantified with ImageJ software.

### Statistical analysis

All statistical analyses were performed with GraphPad Prism 6 (GraphPad Software, San Diego, USA). We did all experiments twice with the three cell lines. Mean values of the two replicates were calculated for each line and used for the further statistical calculations (therefore n = 3). To examine any influence of two independent variables (wt- and dE-variant, induction by Doxy and no induction) or different concentrations of Doxy, on one dependent variable, like DAPI/MAP2/Nestin/Myc/BrdU-positive stained cells, we employed the two-way analysis of variance (two-way ANOVA) with Tukey’s multiple comparisons test. The effect of Doxy on cells overexpressing only rtTA vs. rtTA + TOR1A-wt & -dE was calculated by the Kruskal–Wallis test for multiple comparisons. Results were considered significant at p-values < 0.05.

## Electronic supplementary material

Below is the link to the electronic supplementary material.

Supplementary file1 (XLSX 14 kb)
